# Western Dental Society

**Published:** 1856-06

**Authors:** Henry Barrow


### WESTERN DENTAL SOCIETY.

For the purpose of elevating and perfecting the noble profession of dentistry, by the cultivation of an enlarged liberality of sentiment, by the fostering of an honorable spirit of emulation, and by the full and free interchange of views and opinions, the Professors of Dentistry of the surrounding country and neighboring States, met in the city of St. Louis, on the third day of April, in the large hall of the St. Louis Medical College, and organized the Western Dental Society; the following were the officers elected for the ensuing year:

Dr. Edward Hale, of St. Louis, *President*; Dr. H. E. Peebles, of Lexington. Mo.; 1*st Vice President;* Dr. W. W. Allport, of Chicago, Ill., 2*d Vice President;* Dr. Henry Barrow, of St. Louis, *Recording Secretary;* Dr. C. W.

Spalding, of St. Louis, *Corresponding Secretary;* Dr. A. Blake, of St. Louis, *Treasurer*; Dr. M.. W. Hicks, of Keokuk, Iowa, Dr. H. N. Lewis, of Quincy, Ill., Dr. H. J. B. McKellops, of St. Louis, Dr. S. Dunham, of St. Louis, Dr. Isaiah Forbes, of St. Louis, *Executive Committee.*

A Committee appointed for the purpose, reported a Constitution which was adopted.

The Executive Committee are instructed to procure a certificate and seal, and issue one to each member of the society as a testimonial.

The subject of arsenic, fang filling, and anæsthesia were ably discussed during the session. On motion, it was resolved, that any member, who may have performed operations of a peculiar nature, present the same to the society. The following was unanimously adopted.

*Resolved*, That the thanks of this Society be tendered to the Faculty of the Medical College of St. Louis, for the use of their Hall.

*Resolved*, That when this society adjourn, they adjourn to meet in Chicago on Wednesday the 30th of July. Adopted.

Dr. H. Allport, of Chicago, rose and asked permission to make a few remarks in behalf of his non-resident brethren.

Mr. President. In consequence of the generous treatment we have received from our professional brethren of St. Louis, by participating in social interchange with our city Brethren and the heads of the St. Louis Medical College, in the enjoyment of a glorious banquet, where there was a feast of reason and flow of soul, we met together when the following was unanimously adopted, and we ask permission to have it spread upon the minutes.

*Resolved*, That we, the non-resident dentists express our gratitude and thanks to the dentists of St. Louis, for the hospitable manner and brotherly affection with which they have received and entertained us while in their city.

Permission was granted, and the Society adjourned.

EDWARD HALE, President.

HENRY BARROW, Rec. Sec'y.

It is with pleasure we publish the proceedings of the Western Dental Society, held in St. Louis on 5th of April last. These social gatherings are of vast service to individual members, as well as to the profession itself. We should like very much indeed to be present at the next meeting in Chicago; but have determined, if possible, to meet the general convention in New York 1st of August, and it would be almost impossible to attend both. Let me suggest to the Western Dental Society, to have their next meeting after that at Chicago at Cincinnati, during the next meeting of the Mississippi Association. We then can have a splendid time of it. We will give full use of rooms in the Dental College, or hold sessions together. The Railroad to St. Louis is to be completed before that time. [Ed.

